# Reliability of a Novel High Intensity One Leg Dynamic Exercise Protocol to Measure Muscle Endurance

**DOI:** 10.1371/journal.pone.0163979

**Published:** 2016-10-05

**Authors:** Benjamin Pageaux, Romuald Lepers, Samuele M. Marcora

**Affiliations:** 1 Endurance Research Group, School of Sport & Exercise Sciences, University of Kent at Medway, Chatham Maritime, Kent, United Kingdom; 2 CAPS UMR1093, INSERM, Université Bourgogne Franche-Comté, Dijon, France; University of Rome, ITALY

## Abstract

We recently developed a high intensity one leg dynamic exercise (OLDE) protocol to measure muscle endurance and investigate the central and peripheral mechanisms of muscle fatigue. The aims of the present study were to establish the reliability of this novel protocol and describe the isokinetic muscle fatigue induced by high intensity OLDE and its recovery. Eight subjects performed the OLDE protocol (time to exhaustion test of the right leg at 85% of peak power output) three times over a week period. Isokinetic maximal voluntary contraction torque at 60 (MVC_60_), 100 (MVC_100_) and 140 (MVC_140_) deg/s was measured pre-exercise, shortly after exhaustion (13 ± 4 s), 20 s (P20) and 40 s (P40) post-exercise. Electromyographic (EMG) signal was analyzed via the root mean square (RMS) for all three superficial knee extensors. Mean time to exhaustion was 5.96 ± 1.40 min, coefficient of variation was 8.42 ± 6.24%, typical error of measurement was 0.30 min and intraclass correlation was 0.795. MVC torque decreased shortly after exhaustion for all angular velocities (all P < 0.001). MVC_60_ and MVC_100_ recovered between P20 (P < 0.05) and exhaustion and then plateaued. MVC_140_ recovered only at P40 (P < 0.05). High intensity OLDE did not alter maximal EMG RMS of the three superficial knee extensors during MVC. The results of this study demonstrate that this novel high intensity OLDE protocol could be reliably used to measure muscle endurance, and that muscle fatigue induced by high intensity OLDE should be examined within ~ 30 s following exhaustion.

## Introduction

Endurance performance (i.e. exercise duration > 1 min) is extensively studied in exercise physiology using cycling and/or running exercise (e.g. [1–4]). Despite being close to real competition events by involving the whole-body, the use of cycling and/or running exercise presents some important limitations to understand the role of the central nervous system (CNS) in the regulation of muscle fatigue and endurance performance. Indeed, as whole-body exercise involves greater systemic responses than isolated exercise [5], it is difficult to interpret some specific experimental manipulations aiming to understand CNS processes regulating muscle fatigue and endurance performance (e.g. manipulation of III-IV muscle afferents [6, 7]). Furthermore, due to the need to transfer the participant from the treadmill/bicycle to the ergometer, the true extent of muscle fatigue at exhaustion is underestimated [8], leading to inconclusive results on how peripheral (i.e. fatigue produced by changes at or distal to the neuromuscular junction [9]) and central (i.e. decrease in maximal voluntary activation level [9]) components of muscle fatigue might interact between each other’s (for review see [2, 9]). Therefore, due to the aforementioned limitations, the development of a new exercise model is required to better investigate the CNS processes regulating endurance performance.

Around thirty years ago, Andersen et al. [10] developed a novel exercise model (i.e. one leg dynamic exercise, OLDE) allowing dynamic isotonic contractions of the knee extensor muscles. This exercise model isolates the knee extensor muscles via an active knee extension and passive knee flexion, and due to the reduced muscle mass involved, this exercise is not limited by cardiorespiratory function [11]. Therefore, this model was extensively used to investigate the effect of OLDE on the cardiorespiratory system (e.g. [12]), skeletal muscle physiology (e.g. [13]) but also with patients suffering from cardiorespiratory limitations [14, 15] or for studying mechanisms regulating circulatory response to rhythmic dynamic exercise [6, 16]. More recently, high intensity OLDE has been used to investigate CNS processes involved in the regulation of muscle fatigue and endurance performance [8, 11, 17, 18]. Despite being recently used to investigate muscle endurance, the reliability of high intensity OLDE has not been tested. Reliability can be defined as the consistency of a performance measure, and should be established for any new measurement tool [19, 20]. Furthermore, reliability of a protocol can be used to estimate the sample size required for an appropriate statistical power [20]. The main aim of this study was to establish the reliability of high intensity OLDE as a measure of muscle endurance. Additionally, as the sensitivity of a protocol reflects its ability to detect small changes in performance, we also calculated the smallest worthwhile change as a measure of sensitivity [21].

When performed at high intensity until exhaustion, OLDE has been shown to induce both peripheral and central fatigue [11, 17, 18]. However, as the exercise performed in these studies did not take place on the same ergometer where neuromuscular function was tested, the extent of peripheral and central fatigue remained unclear. To avoid the need to transfer the participant from the exercising ergometer to the dynamometer (to assess muscle fatigue), we recently developed in our laboratory a OLDE protocol on a dynamometer, reducing the time delay between cessation of the exercise and start of neuromuscular testing [8]. In this study, we demonstrated that both peripheral and central fatigue significantly recovered between exhaustion and after three minutes, but also that high intensity OLDE alters cortical and spinal excitability. Previous studies [8, 11, 17, 18] describing muscle fatigue induced by high intensity OLDE focused only on isometric muscle fatigue (i.e. muscle fatigue measured during isometric contractions) and did not describe the extent of isokinetic muscle fatigue (i.e. muscle fatigue measured during isokinetic contractions) and its recovery. Consequently, an additional aim of this study was to describe the isokinetic muscle fatigue and its recovery induced by high intensity OLDE.

## Materials and Methods

### Subjects and Ethical Approval

Eight healthy and moderately active (a minimum of 2 h of aerobic activity per week) adults (mean ± SD; age: 22 ± 2 yrs, height: 171 ± 8 cm, weight: 69 ± 8 kg, 5 males and 3 females) volunteered to participate in this study. None of the subjects had any known mental or somatic disorder. Each subject gave written informed consent prior to the study. Experimental protocol and procedures were approved by the local Ethics Committee of the School of Sport and Exercise Sciences, University of Kent at Medway (Ethic clearance Prop97_2013_14). The study conformed to the standards set by the World Medical Association Declaration of Helsinki “Ethical Principles for Medical Research Involving Human Subjects” (2008). All subjects were given written instructions describing all procedures related to the study.

### Study Protocol

**Fig 1:**
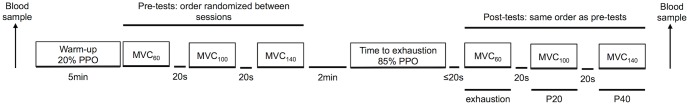
An overview of the study protocol. Angular velocity of the pre and post isokinetic maximal voluntary contraction (MVC) of the knee extensors (KE) tests were randomized between sessions (60-100-140 deg/s, 100-140-60 deg/s or 140-60-100 deg/s). One isometric MVC of the knee flexors was also performed pre and post exercise, 20 s following completion of the last KE MVC. Post tests were performed either shortly after exhaustion (13 ± 4 s), 20 s following exhaustion (P20) or 40 s following exhaustion (P40).

The main aim of this study was to test the reliability of a novel OLDE protocol performed at high intensity (workload fixed at 85% peak power output [22]). Isokinetic muscle fatigue and its recovery up to 40 s post exercise were also measured. Subjects visited the laboratory on four different days. During the first visit, subjects were familiarized with the OLDE protocol (see *One Leg Dynamic Exercise* for more details), and performed after 30 min recovery an incremental test to measure peak power output. After 30 min recovery following the incremental test, subjects were familiarized with neuromuscular testing (see *Neuromuscular Function Tests* for more details) and the time to exhaustion test. As suggested by Andersen et al. [10], torque and electromyographic (EMG) feedback were used to ensure a quick and reliable familiarization to the novel OLDE protocol. Each of the following three visits (reliability sessions) consisted of completion of the time to exhaustion test with neuromuscular testing pre and post-exercise. An overview of these three sessions can be seen in Fig 1.

Each reliability session took place on a Monday, Wednesday and Friday morning at the same time and within the same week. All subjects were given written instructions to drink 35 ml of water per kilogram of body weight, sleep for at least 7 h, refrain from the consumption of alcohol, and avoid any vigorous exercise the day before each visit. Participants were also instructed to avoid any caffeine and nicotine for at least 3 h before testing. Finally, subjects were instructed to consume a set breakfast (2 slices of toast spread with margarine or butter, 250 ml of orange juice, and a banana) 1 h before all testing sessions. At each visit to the lab, subjects were asked to complete a pre-test checklist to ascertain that they had complied with the instructions given to them, and were asked to report any pain or soreness experienced in their leg (to check for the presence of previous session-induced muscle damage). None of our subjects reported leg muscle pain or soreness at the beginning of each session.

### One Leg Dynamic Exercise

#### Model Development

The OLDE protocol and neuromuscular function tests were performed on a Cybex NORM isokinetic dynamometer (CMSi, Computer Sports Medicine Inc., Stoughton, USA). The axis of the dynamometer was aligned with the knee axis, and the lever arm was attached to the shank with a strap. Two shoulder harnesses and a belt across the abdomen limited extraneous movement of the upper body. Full description of the OLDE protocol can be found in Pageaux et al. [8]. Briefly, this protocol allows isolating the knee extensor muscles during a dynamic exercise involving an active isotonic knee extension (from 10 deg to 90 deg, 0 deg = knee fully extended) and a passive knee flexion. The passive flexion angular velocity was set up at 300 deg/s automatically cushioned by the dynamometer for safety purposes. Due to this cushion, the passive knee flexion angular velocity was ~ 180 deg/s. According to a previous study [8], a cadence of 50 contractions per minute (cpm) was chosen (knee extension angular velocity ~ 106°/s). Subjects maintained a cadence of 50 cpm at all visits via the use of a metronome. Power output produced by the subject was controlled according to the formula:

$$P=T\;\times \;\dot{\theta }$$

**Fig 2:**
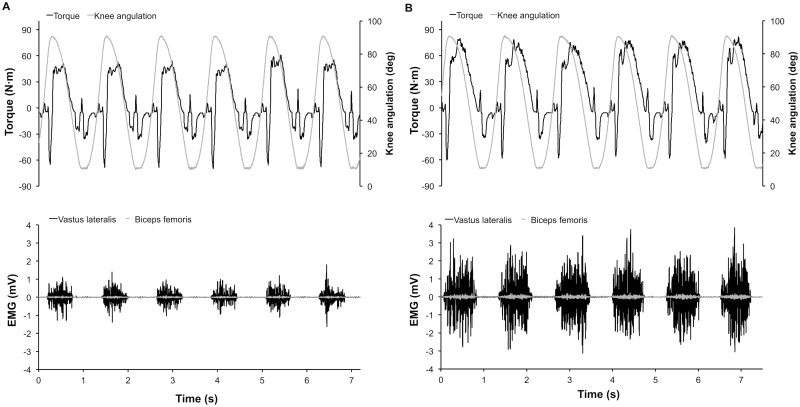
A typical recording of torque, knee angulation, Vastus Lateralis electromyography (EMG) and Biceps Femoris EMG during one leg dynamic exercise with isotonic resistance at 9 N∙m (~ 16.7 W, panel A) and 37 N∙m (~ 68.5 W, panel B).

P corresponds to the power expressed in watt (W), *T* the torque in newton meter (N·m) and $\dot{\theta }$ the angular velocity in rad/s. Typical recordings of torque, position and EMG signals from the Vastus Lateralis (knee extensor) and Biceps Femoris (knee flexor) could be found in Fig 2. Fig 2 presents all signals previously mentioned for an isotonic resistance of 9 N·m (~ 16.7 W, panel A) and 37 N·m (~ 68.5 W, panel B). The inactivity of the Biceps Femoris during the flexion phase confirms that we were successful in creating a protocol on the dynamometer that isolates the knee extensor muscles during dynamic exercise, as in the exercise model originally proposed by Andersen et al. [10].

#### Incremental test

After familiarization, a preliminary OLDE incremental test was performed until exhaustion to measure peak power output. For males, the incremental test started with the isotonic resistance set at 4 N·m (~ 7.4 W) for 1 min, and increased each minute by 3 N·m (~ 4.5 W) to exhaustion. For females, the isotonic resistance was set up at 4 N·m (~ 7.4 W) for 1 min and increased each minute by 2 N·m (~ 3.7 W). Exhaustion was defined as a decrease in cadence below 40 cpm for a duration ≥ 10 s or when the subject voluntarily stopped.

#### Time to exhaustion test

After 5 min warm up at 20% of peak power output, subjects performed a time to exhaustion at 85% of peak power output. Exhaustion was defined as a decrease in cadence below 40 cpm for a duration ≥ 10 s or when the subject voluntarily stopped. Subjects were not aware of the time elapsed during the time to exhaustion test. Verbal encouragements were provided by an experimenter naïve of time to exhaustion during the previous sessions.

### Physiological and perceptual measurements

#### Heart rate

Heart rate was monitored at the end of each minute during the incremental test and every 30 s during all time to exhaustion tests using a heart rate monitor (Polar RS400, Polar Electro Oy, Kempele, Finland). Heart rate was also monitored immediately at exhaustion during the incremental test and the time to exhaustion tests.

#### Blood lactate and glucose concentrations

10 μl samples of capillary blood were taken from the left thumb pre-exercise (before the warm-up) and after the last MVC (post-exercise) to measure blood lactate and glucose concentrations (Biosen, EFK Diagnostics, London, England).

#### Perception of effort

Perception of effort, defined as “the conscious sensation of how hard, heavy, and strenuous exercise is” [23, 24], was measured during the incremental test (at the end of each minute) and during the time to exhaustion tests (at the end of the warm-up and every 30 s) using the 15 points RPE scale (Borg 1998). Standardized instructions for the scale were given to each subject before the warm-up. Briefly, subjects were asked to rate how hard they were driving their leg during the exercise (leg RPE [8, 24, 25]). Subjects were also asked to not use this rating as an expression of leg muscle pain (i.e., the intensity of hurt that a subject feels in his quadriceps muscles only).

#### Leg muscle pain

Leg muscle pain, defined as “the intensity of hurt that a subject feel in his quadriceps muscles only” [26], was measured during the incremental test (at the end of each minute) and during the time to exhaustion tests (at the end of the warm-up and every 30 s) using the Cook scale [26]. Standardized instructions for the scale were given to each subject before the warm-up. Briefly, subjects were asked to rate the feelings of pain specifically in their quadriceps and not to report other pains they may have experienced (e.g., seat discomfort). Subjects were also asked to not use this rating as an expression of perceived effort [24].

#### Electromyography

EMG of the Vastus Lateralis (VL), Rectus Femoris (RF), Vastus Medialis (VM) and Biceps Femoris was recorded with pairs of silver chloride circular (recording diameter of 10 mm) surface electrodes (Swaromed, Nessler Medizintechnik, ref 1066, Innsbruck, Austria) with an interelectrode (center-to-center) distance of 20 mm. Recording sites (belly of each muscle, as distal as possible from the hips when the subject was asked to contract his quadriceps at a knee angle of 10 deg) were then carefully adjusted at the beginning of each testing session (electrode placement was drawn on the skin with permanent marker to ensure reproducibility of the recording site). Low resistance between the two electrodes (< 5 kΩ) was obtained by shaving the skin, and dirt was removed from the skin using alcohol swabs. The reference electrode was attached to the patella of the right knee. Myoelectrical signals were amplified with a bandwidth frequency ranging from 10 Hz to 500 Hz (gain: VL = 500; RF and VM = 1000), digitized on-line at a sampling frequency of 2 kHz using a computer, and stored for analysis with commercially available software (Acqknowledge 4.2 for MP Systems, Biopac Systems Inc., Goleta, USA). Due to the pressure of the thigh on the dynamometer chair, the Biceps Femoris EMG signal quality was impaired (e.g. numerous artefacts, problems with electrodes) and therefore not analyzed.

The EMG signals were filtered with a Butterworth band pass filter (cutoff frequencies 20 and 400 Hz). Then, the root mean square (RMS) of the EMG signal was automatically calculated with the software. During the incremental test, the EMG RMS was averaged for the last 5 EMG bursts of each step (at the end of each minute) and at exhaustion. During the time to exhaustion tests, the EMG RMS was averaged for the last 5 EMG bursts prior each time point measurement (10, 20, 30, 40, 50, 60, 70, 80, 90 and 100% of the time to exhaustion). EMG RMS of each muscle during the time to exhaustion tests was normalized by the maximal EMG RMS of the respective muscle obtained during the pre-exercise KE MVC performed at 100 deg/s. During the KE MVCs, maximal EMG RMS was averaged over a range of 20 deg extension (± 10 deg) around the peak torque.

### Neuromuscular Function Tests

Neuromuscular function tests were performed pre and post-exercise to quantify muscle fatigue. As previous studies documented the extent of isometric muscle fatigue induced by OLDE [8, 11, 17, 18], we chose to focus only on isokinetic muscle fatigue. Therefore, knee extensors (KE) MVCs were performed at 60 (MVC_60_), 100 (MVC_100_) and 140 (MVC_140_) deg/s pre (after the warm-up) and post-exercise (13 ± 4s after exhaustion). Subjects were asked to perform two maximal isokinetic knee extensions at each angular velocity (starting position corresponded to knee angle at 90 deg; range of motion was the same as the OLDE). The highest peak torque value of the two trials was considered, and a 20 s recovery was set between each set of KE MVCs. The order of contractions was randomized between sessions as follow (60-100-140 deg/s, 100-140-60 deg/s or 140-60-100 deg/s) and identical for testing pre and post-exercise of the same session. This randomization allows obtaining a time course of KE MVC torque recovery following the time to exhaustion test at each angular velocity was obtained at a different time point at each session: either shortly after exhaustion (13 ± 4 s after exhaustion), 20 s following exhaustion test (P20) and 40 s following exhaustion test (P40). An overview of timing can be found in Fig 1. Twenty seconds after completion of the last KE MVC, a maximal isometric MVC of the knee flexors was performed (isometric KF MVC). Visual feedback of the torque and strong verbal encouragement were provided for each MVC [please see reference 9 for more details].

#### Mechanical recordings

Torque signal and knee angle signal were recorded using the same dynamometer as for the OLDE (Cybex NORM isokinetic dynamometer, CMSi, Computer Sports Medicine Inc., Stoughton, USA). During the tests a two shoulder harnesses and a belt across the abdomen limited extraneous movement of the upper body. Torque signal and knee angle signal were digitized on-line at a sampling frequency of 1 kHz using a computer, and stored for analysis with commercially available software. Torque signal was filtered prior to data analysis (Butterworth low-pass filter at 100 Hz). Torque signal, knee angle signal and EMG signal were recorded with the same device (MP150, Biopac Systems Inc., Goleta, USA) and analyzed with the same commercially available software (Acqknowledge 4.2 for MP Systems, Biopac Systems Inc., Goleta, USA).

### Statistical Analysis

All data are presented as means ± standard deviation (SD) unless stated. Assumptions of statistical tests such as normal distribution and sphericity of data were checked as appropriate. Greenhouse-Geisser correction to the degrees of freedom was applied when violations to sphericity were present. For reliability statistics, assumptions of homoscedasticity and heteroscedasticity were checked as appropriate. Reliability analysis was conducted following the guidelines provided by Atkinson and Nevill [19]. Our sample size of eight subjects is comparable to previous studies using high-intensity OLDE [8, 11, 17].

#### Incremental test

One way repeated ANOVAs (time: isotime from first to seventh minute and exhaustion) were used to test the time course of EMG RMS for all muscles, leg RPE, leg muscle pain and heart rate. Significant effect of time was explored with planned comparison (1^st^ minute vs other time points, exhaustion vs other time points) adjusted with Holm-Bonferonni correction.

#### Time to exhaustion

One way repeated ANOVA was used to compare time to exhaustion between sessions (S1, S2 and S3). Relative reliability was calculated with the intraclass correlation (ICC) model (3, 1) [27]. Absolute reliability was calculated with the typical error of measurement (the standard deviation of the change scores divided by √2 [28, 29]). Bland and Altman’s 95% limits of agreement were also used (calculated for S1 vs S2, S1 vs S3 and S2 vs S3) as an additional representation of measurement error and to identify the presence of heteroscedasticity [19]. As data were heteroscedastic, both raw data and log transformed Bland and Altman’s plots are presented. Limit of agreement ratio (LOA) was also calculated from the log transformed data as follow: LOA = (1.96 × SDdiff / grand mean) × 100; where “SDdiff” represents the SD of the differences between tests (S1 vs S2, S1 vs S3, S2 vs S3) and “grand mean” represents (mean S1 + mean S2 + mean S3)/3. As time to exhaustion data were heteroscedastic, we also calculated the coefficient of variation (CV) for each subject as follow: CV = 100×(SD of the three measurements)/(mean of the three measurements). Mean CV for all subjects were also calculated. We also calculated the smallest worthwhile change (0.2 × *between subjects SD*) [21].

Time course of normalized EMG RMS for all muscles was analyzed with fully repeated measures 3 (session) x 10 (time: from 10 to 100% of time to exhaustion) ANOVA. Fully repeated measures 3 (session) x 11 (time: warm-up and from 10 to 100% of time to exhaustion) ANOVAs were used to analyze the time course of leg RPE, leg muscle pain, heart rate and cadence. Significant effect of time was explored with planned comparison (10% vs other time points, 100% vs other time points) adjusted with Holm-Bonferonni correction.

Changes in blood lactate and glucose concentration were analyzed with fully repeated measures 3 (session) x 2 (time: pre and post exercise) ANOVA.

As the time to exhaustion did not differ between sessions, only main effects of time are reported for each analysis.

#### Neuromuscular tests

One way repeated ANOVA was used to compare pre-exercise neuromuscular parameters between sessions (S1, S2 and S3). As no pre-exercise (pre) neuromuscular parameters differed between sessions (except EMG RMS RF at 60 deg/s), all pre-exercise parameters (except EMG RMS RF at 60 deg/s) were averaged. Neuromuscular parameters were then analyzed with one-way repeated measures ANOVA (time: pre, exhaustion, P20 and P40). Significant effect of time was explored with planned comparison (pre vs exhaustion, exhaustion vs P20, P20 vs P40) adjusted with Holm-Bonferonni correction. Cohen’s effect size f(V) was also calculated.

Changes in isometric KF MVC torque was analyzed with fully repeated measures 3 (session) x 2 (time: pre and post exercise) ANOVA. As the time to exhaustion did not differ between sessions, only main effects of time are reported.

Significance was set at 0.05 (2-tailed) for all analyses, which were conducted using the Statistical Package for the Social Sciences, version 20 for Mac OS X (SPSS Inc., Chicago, IL, USA). Cohen’s effects size f(V) was calculated with G*Power software (version 3.1.6, Universität Düsseldorf, Germany).

## Results

### Incremental test

Incremental test duration ranged from 7.0 to 11.3 min (9.8 ± 1.6 min). Peak power output ranged from 30 to 56 W (42 ± 11 W). Physiological, psychological and EMG responses to the incremental test are presented in Table 1. All parameters increased over time (all P < 0.009) and planned comparison are presented Table 1.

**Table 1:** Time course of perceptual responses and EMG root mean square (EMG RMS) during the incremental test. EMG RMS was measured for the following muscles: Vastus Lateralis (VL), Rectus Femoris (RF), Vastus Medialis (VM) and the overall knee extensors (KE; sum VL, RF and VM). Data are presented as mean (SD). Isotime corresponds to the time completed by all subjects prior to exhaustion.

	Isotime (min)							
	1	2	3	4	5	6	7	Exhaustion
Perceptual responses								
Leg RPE	8.5^$ $ $^	10.2^$ $ $^**	11.4^$ $^**	12.4^$ $^**	13.4^$ $^**	14.3^$ $^**	15.1^$ $^**	19.8***
*(6–20)*	(1.9)	(2.3)	(2.8)	(3.0)	(3.4)	(3.2)	(3.5)	(0.5)
Leg muscle pain *(0–10)*	0.4^$ $ $^	1.4^$ $ $^*	2.0^$ $ $^**	2.6^$ $^**	3.0^$ $^*	3.7^$^*	4.6^$^**	8.2***
	(0.6)	(1.2)	(1.3)	(1.7)	(1.8)	(2.3)	(2.7)	(2.0)
Heart rate	93.9^$ $ $^	98.5^$ $ $^	105.3^$ $ $^*	110.8^$ $ $^*	117.4^$ $^*	122.1^$^*	128.8^$^**	146.4***
*(beats/min)*	(14.2)	(15.0)	(18.4)	(24.5)	(27.6)	(34.4)	(34.6)	(26.7)
**EMG RMS**								
VL *(mV)*	0.096^$ $^	0.109^$^	0.123^$^	0.127^$^	0.152^$ $^	0.175	0.175	0.231**
	(0.030)	(0.012)	(0.050)	(0.055)	(0.093)	(0.136)	(0.088)	(0.090)
RF *(mV)*	0.073^$^	0.089^$^	0.096	0.096	0.109	0.128	0.128	0.165*
	(0.026)	(0.028)	(0.033)	(0.031)	(0.037)	(0.040)	(0.041)	(0.051)
VM *(mV)*	0.085^$^	0.099^$^*	0.110	0.113	0.126	0.141	0.144	0.196*
	(0.014)	(0.013)	(0.016)	(0.019	(0.026)	(0.037)	(0.032)	(0.041)
KE *(mV)*	0.255^$ $^	0.297^$^*	0.328^$^	0.336^$^	0.387^$^	0.437	0.448*	0.593**
	(0.048)	(0.055)	(0.103)	(0.115)	(0.174)	(0.244)	(0.175)	(0.194)

* significantly different from first minute,

^$^ significantly different from exhaustion,

1 item for P < 0.05, 2 items for P < 0.01 and 3 items for P < 0.001.

### Time to exhaustion tests

#### Reliability of the novel high intensity OLDE protocol

**Fig 3:**
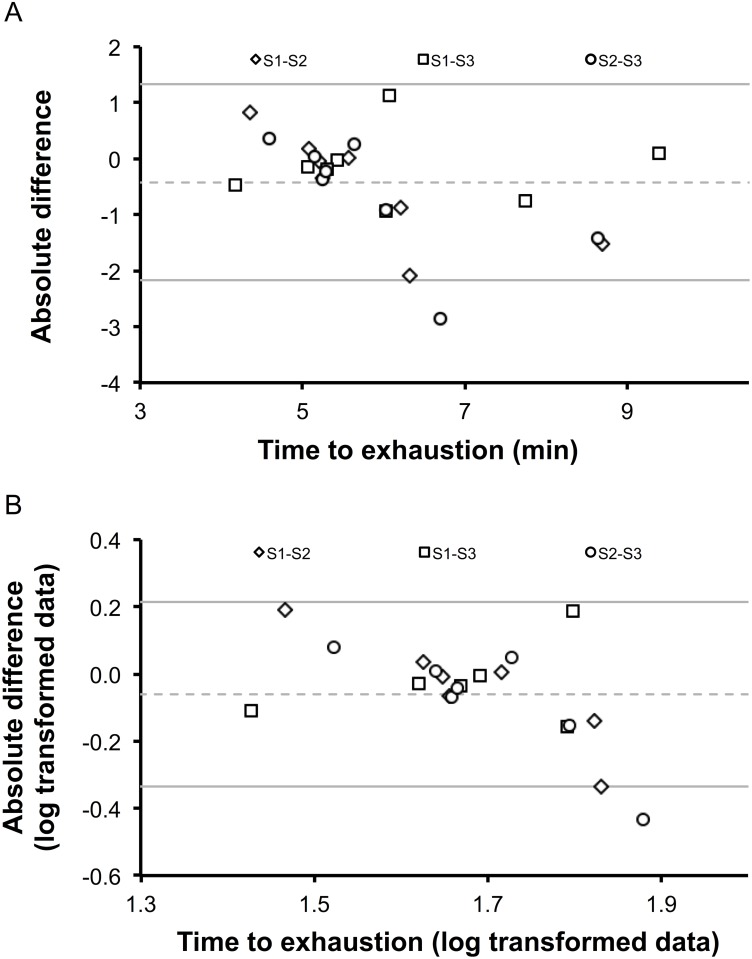
Bland Altman plots (raw data, panel A; log transformed data, panel B) for the time to exhaustion tests. The differences between sessions (S; S1-S2, S1-S3, S2-S3) are plotted against each individual’s mean of the respective two tests. As data were heteroscedastic, limits of agreements ratio (LOA) was also calculated from the log transformed data (LOA = 15.59).

Individual and group time to exhaustion duration are presented Table 2. Time to exhaustion duration ranged from 3.94 to 9.44 min (S1: 6.07 ± 1.71 min, S2: 5.59 ± 0.99 min, S3: 6.23 ± 1.68 min) and did not differ between sessions (P = 0.156). Individual and group CV are presented in Table 2. The ICC was 0.795 (0.493, 0.950). The typical error of measurement was 0.30 min and the smallest worthwhile change was 0.28 min. Bland-Altman plots for raw and log transformed data are presented in Fig 3. LOA was equal to 15.59.

**Table 2:** High intensity one-leg dynamic exercise (85% peak power output) time to exhaustion and coefficient of variation (CV). One-way repeated ANOVA revealed no difference between sessions (p = 0.156). Mean (SD) and individual data are presented.

	Time to exhaustion (min)				
Subjects	Session 1	Session 2	Session 3	Mean	CV (%)
**1**	5.41	5.07	5.44	5.31	3.90
**2**	5.22	5.18	5.40	5.26	2.22
**3**	4.99	5.17	5.13	5.10	1.94
**4**	6.64	5.77	5.50	5.97	9.98
**5**	9.44	7.93	9.34	8.91	9.49
**6**	3.94	4.77	4.41	4.37	9.43
**7**	7.37	5.28	8.13	6.92	21.33
**8**	5.55	5.57	6.49	5.87	9.08
**Mean**	**6.07 (1.71)**	**5.59 (0.99)**	**6.23 (1.68)**	**5.96 (1.40)**	**8.42 (6.24)**

#### Physiological and perceptual measurements

**Fig 4:**
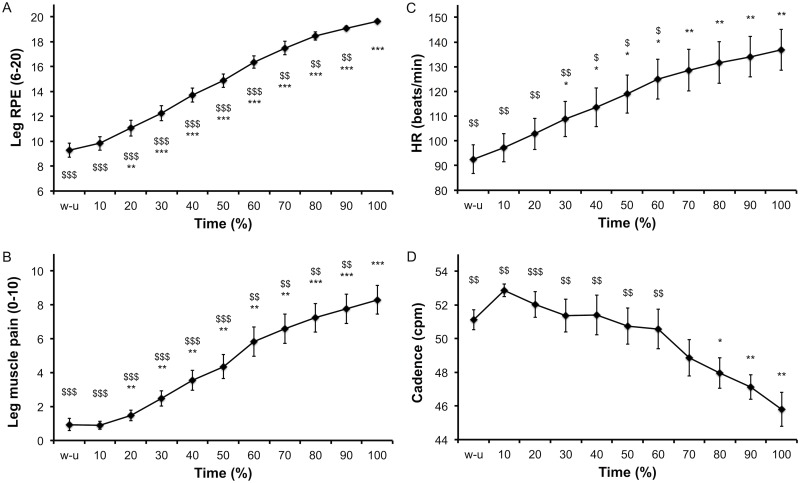
Time course of perceptual responses (panel A and B), heart rate (panel C) and cadence (panel D) during the time to exhaustion tests. Data are presented as main effect of time and mean (SE). * significantly different from 10% and ^$^ significantly different from 100%, 1 item for P<0.05, 2 items for P<0.01 and 3 items for P<0.001.

**Fig 5:**
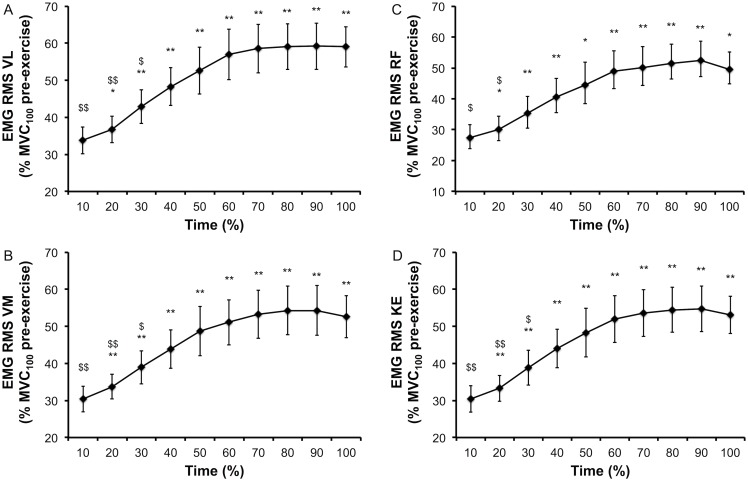
Time course of EMG root mean square (EMG RMS) normalized by the maximum EMG RMS pre-exercise at 100 deg/s (MVC_100_) during the time to exhaustion tests (85% peak power output). EMG RMS was measured for the following muscles: Vastus Lateralis (VL), Rectus Femoris (RF), Vastus Medialis (VM) and the overall knee extensors (KE; sum of VL, RF and VM). Data are presented as main effect of time and mean (SE). * significantly different from 10% and ^$^ significantly different from 100%, 1 item for P < 0.05, 2 items for P < 0.01 and 3 items for P < 0.001.

Physiological, psychological and EMG responses to the time to exhaustion tests are presented Figs 4 and 5. Leg RPE (Fig 4A), leg muscle pain (Fig 4B) and heart rate (HR, Fig 4C) increased over time (all P < 0.001). Cadence during the time to exhaustion decreased over time (P < 0.001). Planned comparisons for these aforementioned parameters are presented Fig 5. EMG RMS of the VL (Fig 5A), VM (Fig 5B), RF (Fig 5C) and the sum of these muscles (Fig 5D) increased over time (all P < 0.001). Planned comparisons for EMG parameters are presented Fig 5. Blood lactate concentration increased (from 1.3 ± 0.5 to 6.0 ± 1.1 mmol/L, P < 0.001) and blood glucose concentration decreased (from 5.3 ± 0.5 to 4.4 ± 0.3 mmol/L, P = 0.001) over time.

### Isokinetic muscle fatigue induced by high intensity OLDE and its recovery

#### Torque and EMG

**Fig 6:**
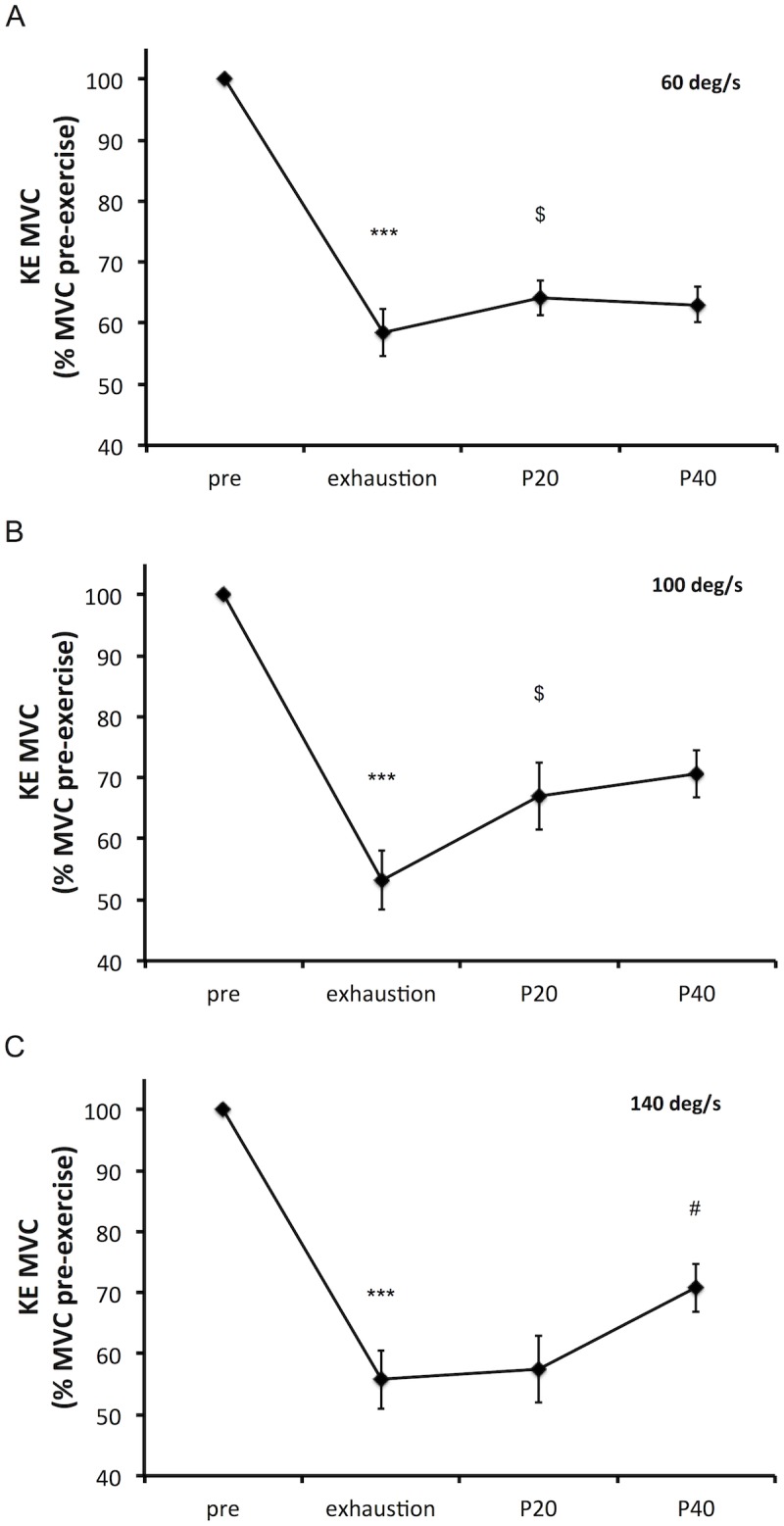
Changes in isokinetic knee extensors maximal voluntary contraction (KE MVC) following the time to exhaustion tests (85% peak power output) and their recovery. Isokinetic KE MVCs were performed at 60 (panel, A), 100 (panel B) and 140 (panel C) deg/s. Isokinetic KE MVCs were measured pre-exercise (pre, average of all three sessions pre-exercise values), shortly after exhaustion (13 ± 4 s after exhaustion), 20 s following exhaustion test (P20) and 40 s following exhaustion test (P40). Data are presented as mean (SE). * significantly different from pre, ^$^ significantly different from exhaustion and ^#^ significantly different from P20, 1 item for P < 0.05 and 3 items for P < 0.001.

**Fig 7:**
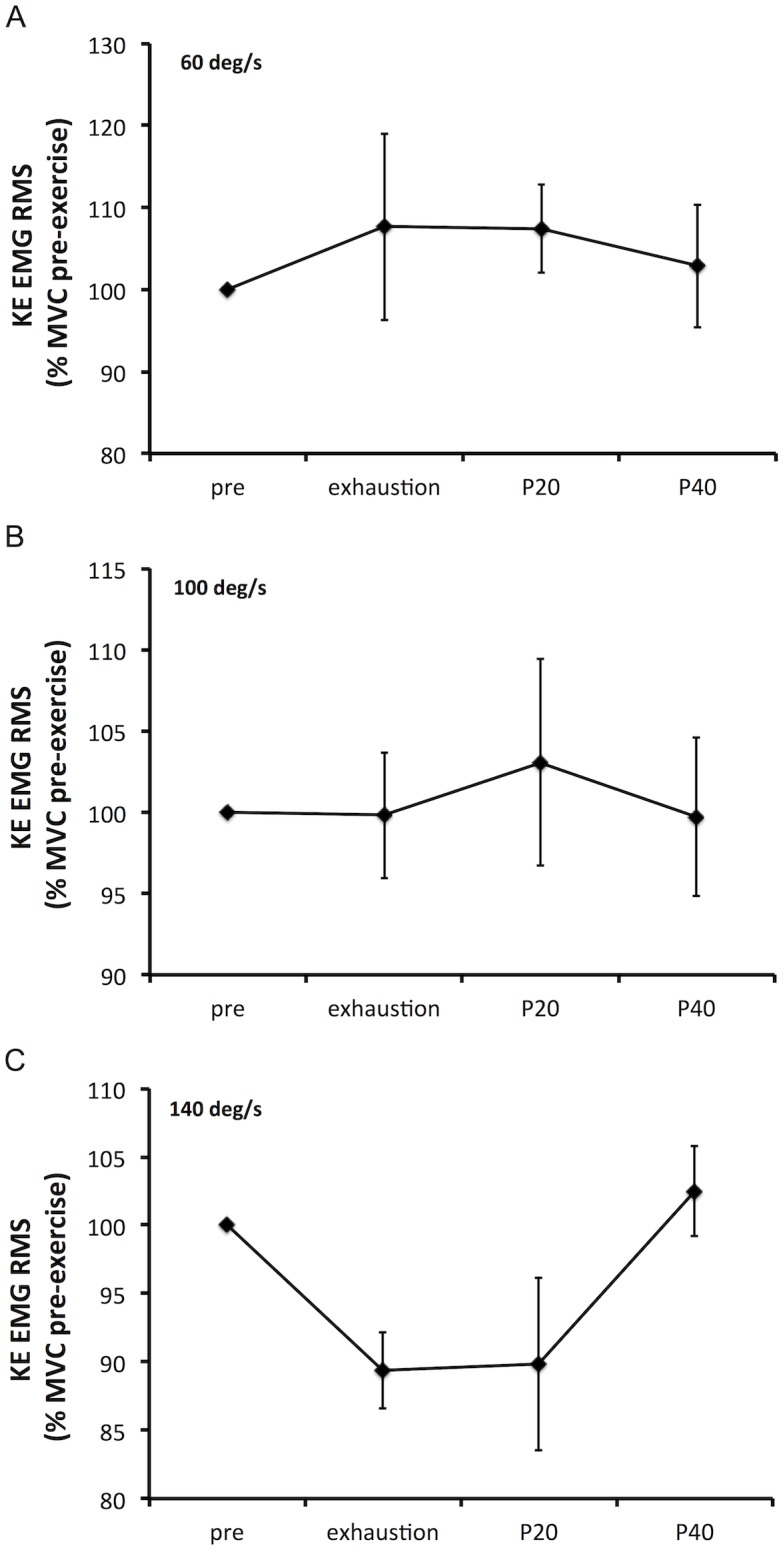
Changes in electromyography root mean square (EMG RMS) during isokinetic knee extensors maximal voluntary contraction (KE MVC) following the time to exhaustion tests (85% peak power output) and during recovery. KE EMG RMS corresponds to the sum of EMG RMS of the following muscles: Vastus Lateralis, Rectus Femoris and Vastus Medialis. Isokinetic KE MVCs were performed at 60 (panel, A), 100 (panel B) and 140 (panel C) deg/s. Isokinetic KE MVCs were measured pre-exercise (pre, average of all three sessions pre-exercise values), shortly after exhaustion (13 ± 4 s after exhaustion), 20 s following exhaustion test (P20) and 40 s following exhaustion test (P40). Data are presented as mean (SE).

Absolute values for KE MVC torques and maximal EMG RMS are presented Table 3. As EMG RMS of the RF muscle at 60 deg/s pre-exercise values significantly differ between sessions, these data were not analyzed. Planned comparisons to explore main effect of time are presented Table 3. Despite a significant main effect of time for the EMG RMS of the RF muscle at 140 deg/s, planned comparison failed to demonstrate a significant difference between times. Changes in KE MVC torque and KE EMG RMS related to baseline are presented Figs 6 and 7. Isometric KF MVC torque did not change over time (75 ± 31 to 73 ± 27 N·m, P = 0.368).

**Table 3:** Time course of isokinetic knee extensors maximal voluntary contraction (KE MVC) and EMG root mean square (EMG RMS) following the time to exhaustion tests (85% peak power output). KE MVCs were performed at 60, 100 and 140 deg/s. Testing was performed pre-exercise (pre, average of all three sessions pre-exercise values), shortly after exhaustion (13 ± 4 s after exhaustion), 20 s following exhaustion test (P20) and 40 s following exhaustion test (P40). As pre-exercise values for the EMG RMS RF at 60 deg/s differ between sessions (P = 0.038), its time course was not analyzed. Planned comparisons failed to demonstrate significant difference between means for EMG RMS RF at 140 deg/s. VL, Vastus Lateralis muscle; RF, Rectus Femoris muscle; VM, Vastus Medialis muscle, KE, knee extensor muscles (sum VL, RF and VM). Data are presented as mean (SD).

	Time				ANOVA	
	Pre	Exhaustion	P20	P40	P-value	Cohen’s effect size f(V)
**60 deg/s**						
KE MVC *(N·m)*	188.7	112.0***	121.3^$^	117.4	< 0.001	3.161
	(57.2)	(41.4)	(41.3)	(33.0)		
EMG RMS VL *(mV)*	0.355	0.402	0.392	0.390	0.341	0.410
	(0.110)	(0.171)	(0.153)	(0.170)		
EMG RMS VM *(mV)*	0.315	0.301	0.346	0.354	0.686	0.179
	(0.117)	(0.182)	(0.134)	(0.163)		
EMG RMS KE *(mV)*	1.038	1.069	1.104	1.087	0.931	0.146
	(0.300)	(0.294)	(0.336)	(0.440)		
**100 deg/s**						
KE MVC *(N·m)*	162.9	86.5***	107.6^$^	111.4	< 0.001	1.648
	(52.7)	(34.2)	(32.8)	(28.6)		
EMG RMS VL *(mV)*	0.392	0.381	0.425	0.361	0.150	0.530
	(0.151)	(0.144)	(0.194)	(0.151)		
EMG RMS RF *(mV)*	0.369	0.359	0.364	0.351	0.978	0.095
	(0.139)	(0.170)	(0.145)	(0.093)		
EMG RMS VM *(mV)*	0.337	0.339	0.335	0.364	0.600	0.301
	(0.151)	(0.132)	(0.150)	(0.179)		
EMG RMS KE *(mV)*	1.038	1.069	1.104	1.087	0.868	0.185
	(0.300)	(0.294)	(0.336)	(0.440)		
**140 deg/s**						
KE MVC *(N·m)*	150.8	84.0**	87.8	106.8^#^	< 0.001	2.092
	(43.8)	(28.9)	(32.7)	(37.1)		
EMG RMS VL *(mV)*	0.414	0.386	0.403	0.428	0.641	0.285
	(0.165)	(0.156)	(0.161)	(0.139)		
EMG RMS RF *(mV)*	0.401	0.327	0.336	0.428	0.031	0.594
	(0.122)	(0.125)	(0.189)	(0.139)		
EMG RMS VM *(mV)*	0.367	0.346	0.351	0.344	0.873	0.182
	(0.141)	(0.165)	(0.157)	(0.117)		
EMG RMS KE *(mV)*	1.182	1.059	1.090	1.200	0.090	0.594
	(0.389)	(0.368)	(0.454)	(0.372)		

* significantly different from pre,

^$^ significantly different from exhaustion

^#^ significantly different from P20,

1 item for P < 0.05, 2 items for P < 0.01 and 3 items for P < 0.001.

## Discussion

The aims of the present study were to assess the reliability of a novel high intensity OLDE protocol to measure muscle endurance, and to describe the isokinetic muscle fatigue induced by high intensity OLDE and its recovery. We demonstrated that our novel high intensity OLDE protocol can be used as a reliable measure of muscle endurance, and that isokinetic muscle fatigue recovers and plateaus within ~ 30 s following exhaustion. Therefore, the novel high intensity OLDE protocol tested in this study might provide an interesting tool to investigate muscle fatigue and muscle endurance.

### Reliability of the novel high intensity OLDE protocol

In the present study, we measured muscle endurance by completion of time to exhaustion tests where the subject has to maintain a fix workload for as long as possible. All time to exhaustion tests lasted less than ten minutes, confirming that OLDE was performed at high intensity. The duration of the time to exhaustion tests in the present study is in accordance with previous studies using the same exercise on a different ergometer [11, 17, 18]. Relative reliability refers to the degree to which individuals maintain their position in a sample with repeated measurements [30]. The ICC value of 0.795 can be interpreted as a questionable reliability (ICC < 0.8), close to the threshold for good reliability (0.8 < ICC < 0.9) [19]. However, as no consensus really exists on threshold to interpret ICC results [31], the practical significance of its value has to be determined with caution by the readers according to their future use of the present protocol. Absolute reliability refers to the degree to which repeated measurements vary for individuals [30]. Traditionally, time to exhaustion tests are known to present a greater CV (CV > 10%) than time trials (i.e. subjects has to perform the greater amount of work possible in a fixed time/distance; CV < 5%) [20]. Interestingly, in our study the CV is below 10%, confirming the great reliability of our novel high intensity OLDE protocol to measure muscle endurance, this despite the small sample size, chosen to be in accordance with previously published studies using the same protocol [8, 11, 17]. This great reliability is confirmed by the typical error of measurement value of 0.30 min, corresponding to 5% of the averaged performance value. Finally, as the typical error of measurement value was slightly above the smallest worthwhile change calculated (0.28 min), it is unlikely that our novel high intensity OLDE protocol can be used to detect small differences in performance.

Interestingly, one of our subjects presented both a CV and a time to exhaustion greater than the other subjects. As both CV and time to exhaustion are known to increase when the intensity of the exercise decreases [20], it is likely that this subject did not reach its true peak power output during the incremental test, and then performed the three time to exhaustion tests at an intensity below 85% of peak power output. This result is of particular importance for future research aiming to manipulate endurance performance using this protocol. Indeed, when the true peak power output is not reached during the incremental test, due to an increase in variability, it might be harder to detect significant changes in muscle endurance. Therefore, in order to better understand the variability in reaching the true peak power output of subjects, further studies should investigate the reliability of the incremental test used in the present study.

During the time to exhaustion tests, all perceptual and physiological measurements increased over time. The increase in heart rate is similar to a previous study using the same exercise on a different ergometer [18]. Furthermore, these authors demonstrated that the respiratory system is not a limiting factor for this exercise. Despite we did not measure the maximum heart rate of our subjects via a typical whole-body incremental test (e.g. cycling), it is clear that a heart rate of ~ 130 beats/min is faraway of the maximum heart rate capacity of our subjects. Therefore, taking all together, these results confirm that high intensity OLDE performed until exhaustion is not limited by the cardiorespiratory system.

### Isokinetic muscle fatigue induced by high intensity OLDE

The second aim of this study was to describe the isokinetic muscle fatigue induced by high intensity OLDE and its recovery. Firstly, the absence of isometric KF MVC torque decrease confirms that our exercise only solicits the knee extensors and does not involve the knee flexors. Secondly, EMG RMS measured during KE MVCs shortly after exhaustion and during the recovery period was not altered by high intensity OLDE, confirming the results of a previous study [8]. Therefore, as a decrease in knee extensors force production capacity can be observed without concomitant changes in EMG signal, our data combined with the data of a previous study [8] suggest that EMG signal cannot be used to investigate dynamic exercise-induced muscle fatigue. The lack of changes in EMG signal is likely to be caused by a potentiation of the maximal evoked muscular wave (M-wave) induced by high intensity OLDE [8]. Finally, according to our hypothesis, isokinetic KE MVC torque quickly recovered and plateaued after exhaustion (within ~ 30 s at 60 and 100 deg/s, and within ~ 50 s at 140 deg/s). This quick recovery in torque production capacity is likely to be associated with recovery in both central and peripheral fatigue. This assumption is supported by one previous study in our laboratory demonstrating that not only peripheral and central fatigue, but also cortical and spinal excitability recovered shortly after exhaustion [8]. Froyd et al. [32] also demonstrated a significant recovery in skeletal muscle function within 1–2 minutes after completion of a one-leg isokinetic time trial performed at high intensity. Taking all together, these results demonstrate that to fully appreciate the extent of neuromuscular alterations induced by high intensity dynamic exercise, assessment of muscle fatigue must be performed within 30 s of cessation of the exercise.

### Conclusion and Perspectives

The results of this study present evidence in favor of this high intensity OLDE protocol to investigate muscle fatigue and muscle endurance. Indeed, this new protocol developed in our laboratory i) presents a lower variability than other high intensity time to exhaustion tests [20], ii) is not limited by the cardiorespiratory system and iii) allows a quick start of neuromuscular testing to fully appreciate the extent of muscle fatigue induced by the exercise. Therefore, it can provide an interesting tool to isolate the cardiorespiratory and neuromuscular effects of various manipulations supposed to play a role in muscle fatigue and performance during high intensity dynamic endurance exercise (e.g. spinal blockade of afferent feedback from the working muscles).
